# Disrupting Circadian Homeostasis of Sympathetic Signaling Promotes Tumor Development in Mice

**DOI:** 10.1371/journal.pone.0010995

**Published:** 2010-06-07

**Authors:** Susie Lee, Lawrence A. Donehower, Alan J. Herron, David D. Moore, Loning Fu

**Affiliations:** 1 Department of Pediatrics/U.S. Department of Agriculture/Agricultural Research Service/Children's Nutrition Research Center, Baylor College of Medicine, Houston, Texas, United States of America; 2 Department of Molecular Virology and Microbiology, Baylor College of Medicine, Houston, Texas, United States of America; 3 Department of Molecular and Cellular Biology, Baylor College of Medicine, Houston, Texas, United States of America; 4 Department of Pathology, Center for Comparative Medicine, Baylor College of Medicine, Houston, Texas, United States of America; 5 Dan L. Duncan Cancer Center, Baylor College of Medicine, Houston, Texas, United States of America; University of Western Ontario, Canada

## Abstract

**Background:**

Cell proliferation in all rapidly renewing mammalian tissues follows a circadian rhythm that is often disrupted in advanced-stage tumors. Epidemiologic studies have revealed a clear link between disruption of circadian rhythms and cancer development in humans. Mice lacking the circadian genes *Period1* and *2* (*Per*) or *Cryptochrome1* and *2* (*Cry*) are deficient in cell cycle regulation and *Per2* mutant mice are cancer-prone. However, it remains unclear how circadian rhythm in cell proliferation is generated *in vivo* and why disruption of circadian rhythm may lead to tumorigenesis.

**Methodology/Principal Findings:**

Mice lacking *Per1 and 2*, *Cry1 and 2*, or one copy of *Bmal1*, all show increased spontaneous and radiation-induced tumor development. The neoplastic growth of *Per*-mutant somatic cells is not controlled cell-autonomously but is dependent upon extracellular mitogenic signals. Among the circadian output pathways, the rhythmic sympathetic signaling plays a key role in the central-peripheral timing mechanism that simultaneously activates the cell cycle clock via AP1-controlled Myc induction and p53 via peripheral clock-controlled ATM activation. Jet-lag promptly desynchronizes the central clock-SNS-peripheral clock axis, abolishes the peripheral clock-dependent ATM activation, and activates *myc* oncogenic potential, leading to tumor development in the same organ systems in wild-type and circadian gene-mutant mice.

**Conclusions/Significance:**

Tumor suppression *in vivo* is a clock-controlled physiological function. The central circadian clock paces extracellular mitogenic signals that drive peripheral clock-controlled expression of key cell cycle and tumor suppressor genes to generate a circadian rhythm in cell proliferation. Frequent disruption of circadian rhythm is an important tumor promoting factor.

## Introduction

Disruption of circadian rhythm increases spontaneous and carcinogen-induced mammary tumors in rodents [1], [2], [3], [4], [5], [6]. Epidemiological studies have revealed that human night-shift workers show an increased risk of breast, colon, lung, endometrial and prostate cancer, hepatocellular carcinoma and non-Hodgkin's lymphoma [7], [8], [9], [10], [11], [12]. Loss of circadian rhythm is also associated with accelerated tumor growth in both rodents and human cancer patients [13], [14], [15]. These findings raise the question of how circadian dysfunction increases the risk of cancers.

Circadian rhythms in mammals are generated by an endogenous clock composed of a central clock located in the hypothalamic suprachiasmatic nucleus (SCN) and subordinate clocks in all peripheral tissues. The SCN clock responds to external cues and drives peripheral clocks via circadian output pathways. Both the central and peripheral clocks are operated by feedback loops of circadian genes, including *Bmal1*, *Clock*, *Period* (*Per1-3*) and *Cryptochrome* (*Cry1* and *2*). *Bmal1* and *Clock* encode bHLH-PAS transcription factors that heterodimerize and bind to E-boxes in gene promoters to activate *Per* and *Cry* transcription, whereas *Per* and *Cry* encode repressors of BMAL1/CLOCK. The alternating activation and suppression of the BMAL1-driven positive loop and the PER/CRY-controlled negative loop result in a circadian oscillation of the molecular clock [16], [17], [18].

The molecular clock regulates clock-controlled genes (CCGs) to control tissue/organ function. Most CCGs follow tissue-specific expression patterns. Only a small group of CCGs, which include key cell cycle regulators and tumor suppressors, are expressed in all tissues studied. Such circadian control leads to the coupling of cell proliferation with key tissue functions *in vivo* [19], [20], [21], [22], [23], [24]. Disruption of circadian rhythm in cell proliferation is frequently associated with tumor development and progression in mammals [4], [5], [12], [25], [26], [27], [28].

Both positive and negative loops of the molecular clock are involved in cell cycle control. For example, BMAL1 suppresses proto-oncogene *c-myc* but stimulates the tumor suppressor *Wee1* [19], [22], [29], CRY2 indirectly regulates the intra S-check point [30], [31], and PER1 directly interacts with ATM in response to γ-radiation *in vitro* [32]. In mice, mutation in *Per*2 leads to deregulation of DNA-damage response and increased neoplastic growth [19], [24], [29]. In humans, deregulation or polymorphism of *Per1*, *Per2*, *Cry2*, *Npas2* and *Clock* is associated with acute myelogenous leukemia, hepatocellular carcinoma, breast, lung, endometrial and pancreatic cancers, and non-Hodgkin's lymphoma [12], [33], [34], [35], [36], [37], [38], [39], [40], [41].

However, peripheral clock-controlled gene expression is not sufficient to generate and maintain the circadian rhythm of cell proliferation because G1 cell cycle progression in normal somatic cells is strictly controlled by extracellular mitogenic signals [42]. *In vivo*, these signals include hormones, growth factors, cytokines and neurotransmitters that follow cyclic changes over a 24 hour period since they are either directly released from circadian output pathways or encoded by CCGs in peripheral tissues [43], [44], [45]. We hypothesize that cell proliferation *in vivo* is paced by both central and peripheral clocks. The central clock-controlled mitogenic signals simultaneously actives the cell cycle and peripheral clocks leading to a circadian coupling of cell cycle and tumor suppressor gene expression. Disruption of circadian rhythm promotes tumor development due to, at least in part, loss of the homeostasis of cell cycle control.

To test our hypothesis, we studied the role of circadian homeostasis of the sympathetic nervous system (SNS) in tumor suppression. The central clock generates a robust circadian rhythm in SNS signaling via direct and indirect targeting of the presympathetic neurons located in the hypothalamic autonomic paraventricular nucleus [43]. *In vivo*, the SNS controls all peripheral tissues by releasing the hormones epinephrine and norepinephrine that target adrenergic receptors (ADRs) on the cell membrane [46]. Norepinephrine is directly released from postganglionic sympathetic neurons, whereas epinephrine is released from preganglionic sympathetic neuron-controlled chromaffin cells located in the adrenal medulla [46]. We have previously reported that all circadian gene-mutant mice studied show hyperplastic growth of osteoblasts in bone, and that β-adrenergic receptor (ADRβ) activation leads to *Per* and *Ap1* induction in primary osteoblasts. In addition, AP1 directly activates *c-myc*, whereas BMAL/CLOCK prevents *myc* over-expression [29]. Since the induction of *Per* leads to peripheral clock activation [47] and that the activation of *c-myc* initiates cell proliferation [48], SNS signaling could be a circadian time cue for both cell cycle and peripheral clocks. Therefore, SNS dysfunction may disrupt the circadian homeostasis of cell cycle control in peripheral tissues.

Here we report that all circadian gene-mutant mouse models studied are cancer-prone and that hyperplastic growth of *Per*-mutant somatic cells is dependent upon extracellular mitogens. Rhythmic SNS signaling plays a key role in pacing cell proliferation rate in peripheral tissues by simultaneously activating *Ap1*-c-myc and ATM-p53 signaling. However, activation of *Ap1* by SNS signaling is independent of the peripheral clock while activation of ATM is peripheral clock-dependent. Disruption of circadian rhythm desynchronizes the central clock-SNS-peripheral clock axis, suppresses peripheral clock function and abolishes peripheral clock-dependent ATM activation, leading to *myc* oncogenic activation and increased incidence of tumors in wild-type mice. Our studies identify a previously unknown molecular pathway that links disruption of circadian rhythm with oncogenesis and demonstrate that tumor suppression *in vivo* is a clock-controlled physiological process but not a non-clock function of a specific circadian gene.

## Results

### Tumor Suppression *in vivo* Is Not a Cell-autonomous Function

**Figure 1:**
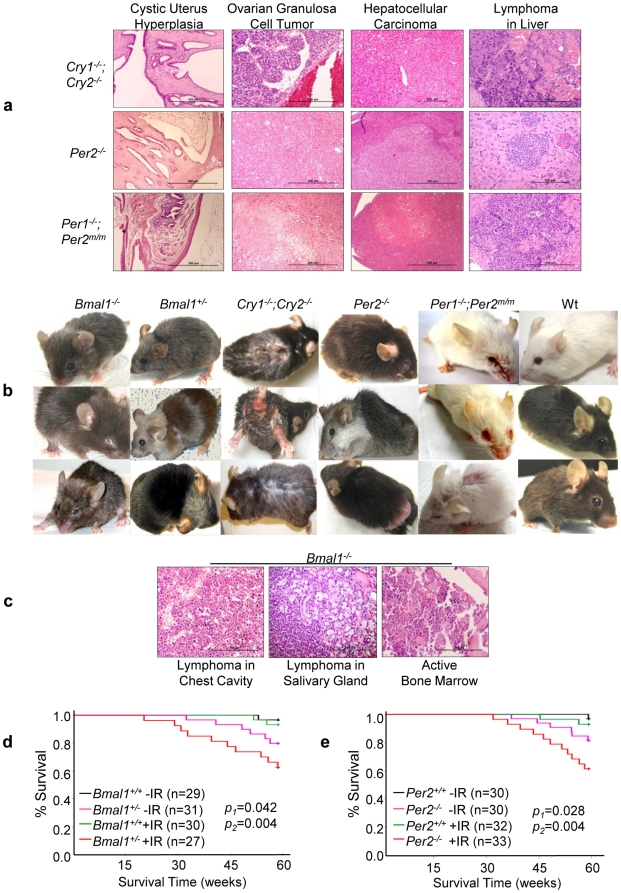
Circadian Gene-mutant Mice Are Cancer-prone. (***a***) Representative histological slides of cystic hyperplasia of the uterus, spontaneous ovarian and liver tumors, and lymphoma in the livers of *Cry*- and *Per*-mutant mice. (***b***) All mutant mouse models studied show aging phenotypes on their external appearance after exposure to a 4 Gy sublethal γ-radiation at 6 weeks of age including hair graying, alopecia, ruffled fur, skin lesions, cataracts and eye inflammation, hunchback postures, body weight changes, and sluggish activities. The ages of irradiated mice shown in (***b***) are: *Bmal1^−/−^* mice at 3–6 months of age, *Bmal1^+/−^* mice at 6–8 months of age, *Cry1^−/−^*;*Cry2^−/−^* mice at 3–6 months of age, *Per2* ^−/−^ mice at 6–8 months of age, *Per1^−/−^*;*Per2^m/m^* mice at 6–8 months of age, and wt littermates at 6–8 months of age. (***c***) Representative histological slides show lymphoma in the chest cavity of a 20-week old and the salivary gland of a 36-week old irradiated *Bmal1^−/−^* mouse, and the active bone marrow in a 40 week-old irradiated *Bmal1^−/−^* mouse that also displayed aging phenotype on the external appearance. The Kaplan-Meier survival curves of (***d***) *Bmal1^+/−^* and (***e***) *Per2^−/−^* mice (−IR: untreated, +IR: irradiated, *p*1: untreated wt vs. untreated mutant littermates, and *p*2: irradiated wt vs. irradiated mutant littermates).

We found that when kept in 24 hour alternating light-dark conditions (24hr LD cycles), mice deficient in *Bmal1* (*Bmal1^+/−^*), *Cry1* and *Cry2* (*Cry1^−/−^*;*Cry2^−/−^*), *Per1* and *Per2* (*Per1^−/−^*;*Per2^m/m^*) or *Per2* alone (*Per2^−/−^*) were all cancer-prone. About 10–15% of *Bmal1^−/−^* mice also showed hyperplasia of salivary glands in spite of a significant reduction in the size of other major organ systems due to aggressive aging [49]. *Bmal1^+/−^*, *Cry*- and *Per*-mutant mice all showed increased risk of ulcerative dermatitis and hyperplasia in the salivary gland, preputial gland, liver and uterus as well as spontaneous lymphoma, liver and ovarian tumor development, although spontaneous tumors in *Cry*-mutants were mostly identified after 50 weeks of age, later than that of *Per1^−/−^*;*Per2^m/m^* and *Per2^−/−^* mice (Fig. 1a, Table 1, Fig. S1a and data not shown).

**Table 1:** Summary of the Causes of Death for Circadian Gene-mutant Mice.

Genotype	1Lymphoma	Osteo-	2Liver	Angio-	3Ovarian	4Uterus/SV•	5Renal	6Ulcerative	7 °Aging	Observation	*p* Value
		sarcoma	Tumor	sarcoma	Tumor	Hyperplasia	Failure	Dermatitis		Period (wks)	
**Untreated**											
†Wt (n = 90)	5.56%	0	1.11%	2.22%	0	∧1.11/6.67%	0	1.11%	0	80	
*Bmal1^+/−^* (n = 31)	12.90%	0	3.23%	0	3.23%	∧3.23/12.90%	3.23%	0	°6.46%	80	= 0.0012
*Bmal1^−/−^* (n = 23)	0	0	0	0	0	4.35%	*4.35/13.04%	0	°65.22%	40	NA
*Per2^−/−^* (n = 30)	13.33%	0	6.67%	0	3.33%	13.33%	3.33%	3.33%	0	80	= 0.0009
*Cry1^−/−^*;*Cry2^+/+^* (*n* = 20)	10.0%	0	5.0%	0	5.0%	15.0%	5.0%	10.0%	0	60	= 0.0003
*Cry1^−/−^*;*Cry2^−/−^* (n = 30)	13.33%	0	6.67%	0	3.33%	20%	0	23.33%	°3.33%	80	= 0.0009
*Per1^−/−^*;*Per2^m/m^* (n = 40)	17.5%	0	10%	0	2.5%	10%	*2.5%	5%	°2.5%	80	= 0.0002
**Irradiated**											
†Wt (n = 88)	10.23%	0	2.28%	4.55%	1.14%	6.82%	0	4.55%	0	80	
*Bmal1^+/−^* (n = 27)	33.33%	0	7.41%	0	3.7%	14.81%	3.7%	3.7%	°14.81%	80	= 0.0007
*Bmal1^−/−^* (n = 24)	8.33%	0	0%	0	0	0	*4.17/16.67%	[Table-fn nt108] a4.17%	°83.33%	40	NA
*Per2^−/−^* (n = 33)	33.33%	3.03%	9.09%	0	6.06%	12.12%	3.03%	[Table-fn nt108] a3.03/6.06%	0	80	<0.0001
*Cry1^−/−^*;*Cry2^+/+^* (*n* = 21)	23.81%	4.76%	9.52%	0	9.52%	14.29%	9.52%	[Table-fn nt108] a4.76/14.29%	0	60	<0.0001
*Cry1^−/−^*;*Cry2^−/−^* (n = 30)	23.33%	3.33%	13.33%	0	6.67%	20%	3.33%	[Table-fn nt108] a10/26.67%	°3.33%	80	<0.0001
*Per1^−/−^*;*Per2^m/m^* (n = 47)	36.17%	2.13%	17.02%	0	6.38%	17.02%	4.26%	10.64%	°2.13%	80	<0.0001
**Irradiated/Shifted**											
†Wt (n = 54)	27.78%	3.70%	7.41%	3.70%	3.70%	∧7.41/16.67%	*7.41/16.67%	12.96%	°1.85%	80	
*Bmal1^−/−^* (n = 26)	7.69%	0	0	0	0	0	*3.85/15.39%	3.85%	°84.62%	40	NA
*Per2^−/−^* (n = 35)	40.0%	5.71%	14.28%	0	8.57%	20.0%	*5.71/14.28%	22.86%	°2.86%	80	= 0.508
*Cry1^−/−^*;*Cry2^−/−^* (n = 33)	36.36%	6.06%	18.18%	3.03%	9.09%	∧6.06/30.30%	*6.06/15.15%	[Table-fn nt108] a12.12/36.36%	°3.03%	80	= 0.0967
*Per1^−/−^*;*Per2^m/m^* (n = 49)	38.78%	4.08%	24.49%	0	10.2%	∧4.08/28.57%	*6.12/14.29%	[Table-fn nt108] a2.04/20.41%	°2.04%	80	= 0.0673

1 Most are B-type lymphomas.

2 Hepatocellular adenoma and adenocarcinoma.

3 Ovarian granulosa cell tumor.

4 Cystic Hyperplasia.

5 Renal hydronephrosis and cystic renal dysplasia with severe hydronephrosis.

6 Ulcerative necrotizing dermatitis and ulcerative dermatitis with cellulitis and fibrosis, and.

7 Aging: untreated *Bmal1^−/−^* mice had an average life span of 35 weeks due to severe aging phenotypes, which was reduced to 27 weeks after irradiation.

a Some irradiated/shifted *Cry1^−/−^*;*Cry2^−/−^*, *Per1^−/−^*;*Per2^m/m^* and wt mice showed similar aging phenotypes as *Bmal1^−/−^* mice and died at 80 weeks of age or older. Some *Per*- and *Cry*-mutant and irradiated/jetlagged wt mice developed more than one type of tumors. Other causes of death include lipoma, pancreatic, intestinal and kidney tumors in irradiated/shifted mice, and malnutrition of *Bma1l^+/−^*, *Cry1^−/−^*;*Cry2^−/−^* and *Per1^−/−^*;*Per2^m/m^* mice due to overgrowth of teeth that prevents food-intake.

*p* Value: comparison of the number of cases of death caused by osteosarcoma, liver and ovarian tumors, and severe hyperplasia of reproductive organs in each mutant mouse model with wt controls in each category (Students' *t*-test).

† Wt mice used for all survival curve studies.

^•^SV: seminal vesicle.

*% mice died due to renal failure.

∧ % mice sacrificed due to severe hyperplasia of uterus or enlargement of seminal vesicles (≥20% of total body weight) causing immobility and lack of food intake.

a % mice sacrificed due to severe ulcerative dermatitis on ≥10% of total body surface.

° % mice sacrificed due to aggressive aging resembling *Bmal1^−/−^* mice.

A sublethal dose of γ-Radiation induced premature aging on the external appearance of all circadian gene-mutant mouse models studied and further increased incidence of tumor and hyperplasia as well as ulcerative dermatitis in *Bmal1^+/−^*, *Per*- and *Cry*-mutant mice (Fig. 1b, Table 1 and Fig. S1b). About 8% of irradiated *Bmal1^−/−^* mice also developed lymphoma despite an average lifespan of 27 weeks due to a further accelerated rate of aging (Fig. 1c and Table 1). Irradiated *Bmal1^+/−^* mice showed a similar rate of tumor development as did irradiated *Per2^−/−^* mice (Fig. 1d–e and Table 1). Since all circadian gene-mutant mouse models show increased sensitivity to γ-radiation, we conclude that the molecular clock functions in tumor suppression *in vivo*.

**Figure 2:**
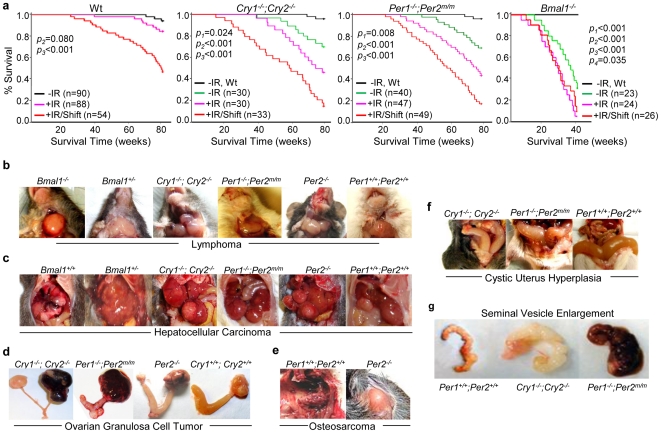
Disruption of Circadian Rhythm Promotes Tumor Development in Wild-type Mice. (***a***) The Kaplan-Meier survival curves of wt, *Cry1^−/−^*;*Cry2^−/−^*, *Per1^−/−^*;*Per2^m/m^* and *Bmal1^−/−^* mice (−IR: untreated, +IR: irradiated, +IR/Shift: irradiated and jet-lagged, *p*1: untreated wt vs. untreated mutant mice, *p*2: untreated wt mice vs. irradiated mice, *p*3: untreated wt mice vs. irradiated/jet-lagged mice, and *p*4: untreated vs. irradiated *Bmal1^−/−^* mice). Median survival times in weeks (95% CI) are 79.7 weeks (79.2–80.1) for untreated, 78.6 weeks (77.5–79.7) for irradiated and 67.9 weeks (63.3–72.5) for irradiated/jet-lagged wt mice, 76.9 weeks (73.9–80.0) for untreated, 69.3 weeks (64.4–74.2) for irradiated and 54.9 weeks (47.8–62.0) for irradiated/jet-lagged *Cry1^−/−^*;*Cry2^−/−^* mice, 74.8 weeks (71.8–77.8) for untreated, 67.1 weeks (62.5–71.7) for irradiated and 56.1 weeks (51.0–61.2) for irradiated/jet-lagged *Per1^−/−^*;*Per2^m/m^* mice, and 35.5 weeks (30.73–40.1) for untreated, 27.3 weeks (23.3–31.4) for irradiated and 28.5 weeks (24.4–32.5) for irradiated/jet-lagged *Bmal1^−/−^* mice. Representative pictures of (***b***) lymphomas in the salivary glands of irradiated/jet-lagged *Bmal1^−/−^*, *Bmal1^+/−^*, *Cry1^−/−^*;*Cry2^−/−^*, *Per2* ^−/−^, *Per1^−/−^*;*Per2^m/m^* and wt (*Per1^+/+^*;*Per2^+/+^*) mice, (***c***) hepatocellular carcinomas in irradiated/jet-lagged *Bmal1^+/−^*, *Cry1^−/−^*;*Cry2^−/−^*, *Per1^−/−^*;*Per2^m/m^*, *Per2* ^−/−^ and wt (*Bmal1^+/+^* and *Per1^+/+^*;*Per2^+/+^*) mice, (***d***) ovarian granulosa cell tumors in irradiated/jet-lagged *Cry1^−/−^*;*Cry2^−/−^*, *Per1^−/−^*;*Per2^m/m^*, *Per2* ^−/−^ and wt (*Cry1^+/+^*;*Cry2^+/+^*) mice, (***e***) osteosarcoma growing out from the spine into the chest cavity of an irradiated/jet-lagged wt (*Per1^+/+^*;*Per2^+/+^*) mouse and on the back of an irradiated/jet-lagged *Per2^−/−^* mouse, (***f***) severe cystic hyperplasia of the uterus in irradiated/jet-lagged *Cry1^−/−^*;*Cry2^−/−^*, *Per1^−/−^*;*Per2^m/m^* and wt (*Per1^+/+^*;*Per2^+/+^*) mice, and (***g***) seminal vesicles from an untreated 60-week old wt (*Per1^+/+^*;*Per2^+/+^*) mouse and age-matched irradiated/jet-lagged *Cry1^−/−^*;*Cry2^−/−^* and *Per1^−/−^*;*Per2^m/m^* mice.

We then examined the role of the central clock in tumor suppression by studying the effects of jet-lag (an 8hr phase-advance followed by an 8hr phase-delay in the onset of the light period every 3 days) on tumor development in mice. We found that jet-lag further increased and hastened tumor development in wt, *Cry*- and *Per*-mutant mice and induced pancreatic, kidney and intestinal tumors in mutant mice. However, jet-lag did not significantly change the overall survival and tumor development of irradiated *Bmal1^−/−^* mice that were deficient in responding to circadian light cues in the central clock (Table 1, Fig. 2a, Fig. S1c and data not shown) [50]. Jet-lag also significantly changed tumor spectrum and induced osteosarcoma, liver and ovarian tumors, hyperplasia of the salivary gland, liver and uterus as well as severe seminal vesicle enlargement in wt mice (Table 1. Fig. 2b–g, Fig. S1d–l and data not shown). In addition, although when kept 24hr LD cycles, female circadian gene-mutant mice showed an earlier onset and a higher tumor incidence compared to male mutant mice, we did not found a significant gender difference in total tumor incidence and the time of tumor onset among irradiated and jet-lagged male and female mutant mice (data not shown).

### Sympathetic Signaling Is a Circadian Time Cue

**Figure 3:**
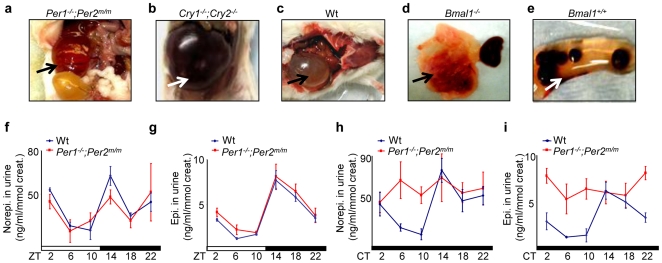
Circadian Control of Sympathetic Signaling. (***a–e***) Jet-lag induces fatal kidney failure in mice. The arrows indicate (***a***) severe cystic renal dysplasia with hydronephrosis of the remaining kidney (>20 times enlarged) in an irradiated and jet-lagged *Per1^−/−^*;*Per2^m/m^* mouse, (***b***) the remaining failed kidney in an irradiated and jet-lagged *Cry1^−/−^*;*Cry2^−/−^* mouse, (***c***) the remaining failed kidney in an irradiated and jet-lagged wt mouse, (***d***) the failed kidney next to the other apparently normal kidney from an untreated *Bmal1^−/−^* mouse, and (***e***) the remaining failed kidney with kidney stones accumulated inside in an irradiated and jet-lagged wt mouse (*Bmal1^+/+^*). (***f–i***) Summaries of urinary norepinephrine and epinephrine levels in wt and *Per1^−/−^*;*Per2^m/m^* mice in (***f–g***) 24hr LD (ZT) and (***h–i***) DD (CT) cycles detected from 3 to 6 independent experiments (ZT: Zeitgeber Time, with light on at ZT0 and off at ZT12; CT: Circadian Time, with CT0 as the beginning of the subjective day and CT12 as the beginning of the subjective night) (±SEM).

We found that chronic jet-lag disrupted the circadian rhythm in urine levels of creatinine and creatinine kinase and significantly increased the risk of renal failure in mice (Table 1 and data not shown). Mice that displayed fatal renal failure usually had only one kidney left at the time of pathological examination which completely lost filtration function due to severe hydronephrosis leading to pressure atrophy and loss of the renal cortex (Fig. 3a–e). Since renal failure has a strong correlation with sympathetic dysfunction [51], this finding indicates that jet-lag disrupts the homeostasis of multiple circadian output pathways including the SNS. To test this hypothesis, we first studied urine catecholamine levels in wt and *Per1^−/−^*;*Per2^m/m^* mice. We found that in 24hr LD cycles, both wt and *Per1^−/−^*;*Per2^m/m^* mice showed a two-peak oscillation in norepinephrine and a one-peak oscillation in epinephrine levels in urine (Fig. 3f–g). In constant darkness (24hr DD cycles), the urinary catecholamine levels still oscillated with a similar pattern as in 24hr LD cycles in wt mice, but were significantly elevated and arrhythmic in *Per1^−/−^*;*Per2^m/m^* mice (Fig. 3h–i). We conclude that one of the urine norepinephrine peaks is generated by central clock-controlled postganglionic sympathetic neuron activity, while the other is generated by preganglionic sympathetic neuron-controlled chromaffin cells that also rhythmically release epinephrine. Since the central clock in *Per1^−/−^*;*Per2^m/m^* mice is driven by environmental light in LD cycles but is not functional in DD cycles [52], these mice could maintain a circadian rhythm of urinary catecholamine levels in LD cycles but not in DD cycles.

**Figure 4:**
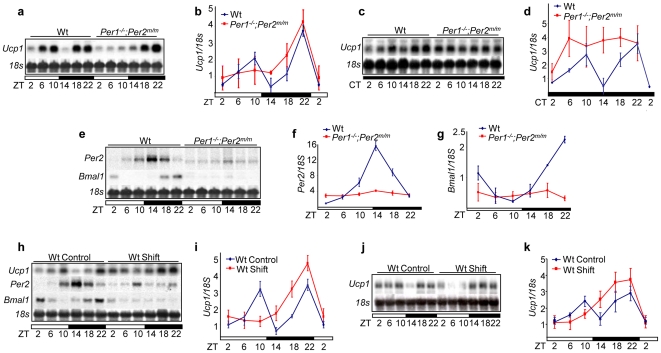
Central Clock-controlled Sympathetic Signaling is a Key Central-peripheral Timing Mechanism. (***a***) Northern blot of *Ucp1* mRNA expression in wt and *Per1^−/−^*;*Per2^m/m^* BAT in 24hr LD cycles. (***b***) A summary of three independent Northern blot analyses described in (***a***) (±SEM). (***c***) *Ucp1* mRNA expression in wt and *Per1^−/−^*;*Per2^m/m^* BAT in 24hr DD cycles. (***d***) A summary of three independent Northern blot analyses described in (***c***) (±SEM). (***e***) *Per2* and *Bmal1* expression in wt and *Per1^−/−^*;*Per2^m/m^* BAT in 24hr LD cycles. Summaries of (***f***) *Per2* and (***g***) *Bmal1* mRNA expression in 24hr LD cycles from three independent experiments (±SEM). (***h***) *Per2*, *Bmal1* and *Ucp1* mRNA expression in BAT from untreated (Control) wt mice and wt mice treated with one cycle of jet-lag (Shift). (***i***) A summary of three independent Northern blot analyses described in (***h***) (±SEM). (***j***) *Ucp1* mRNA expression in BAT from age-matched control wt mice and wt mice treated with 10 months of chronic jet-lag. (***k***) A summary of *Ucp1* mRNA expression from three independent Northern blot analyses described in (***j***).

We then studied whether the SNS rhythmically activates gene expression in peripheral tissues. We found that among various SNS targets studied, *Ucp1* mRNA, which is expressed in brown adipose tissue under the control of a *CRE* site in *Ucp1* promoter [53], could be used as a molecular marker to analyze the activities of both central and peripheral clocks. In 24hr LD cycles, *Ucp1* mRNA showed a two-peak expression in wt BAT, with the first peak appearing at ZT10 and the second at ZT22. In *Per1^−/−^*;*Per2^m/m^* BAT, only the ZT22 peak was observed (Fig. 4a–b). In 24hr DD cycles, *Ucp1* mRNA still followed a two-peak expression in wt BAT, but was arrhythmic and high in *Per1^−/−^*;*Per2^m/m^* BAT (Fig. 4c–d). In wt mice, the activation of CREB peaked between ZT10 and ZT18 (Fig. S2a–b). Loss of function in *Per1* and *Per2* had no effect on CREB activation induced by ADRβ signaling (Fig. S2c), but abolished the peripheral clock activity as shown by lacking a rhythmic expression of *Per2* and *Bmal1* mRNAs in *Per1^−/−^*;*Per2^m/m^* BAT (Fig. 4e–g). Thus, we conclude that the ZT10 peak of *Ucp1* mRNA is generated by a collaborative activity of the central and peripheral clocks, whereas, the ZT22 peak of *Ucp1* mRNA is exclusively controlled by the central clock.

We next examined the effect of jet-lag on *Ucp1* mRNA expression in wt mice on the second day after one cycle of jet-lag. We found that although the ZT22 peak of *Ucp1* mRNA was found in both control and jet-lagged mice, the ZT10 peak of *Ucp1* mRNA was absent in jet-lagged mice, which correlated with a lack of rhythmic expression of *Per2* and *Bmal1* mRNAs in the same BAT (Fig. 4h–i, Fig. S2d–e). We then studied *Ucp1* expression in wt mice treated with chronic jet-lag for 10 months and found that *Ucp1* mRNA expression followed a similar pattern in these mice compared to wt mice treated with only one cycle of jet-lag (Fig. 4j–k). Together, our studies demonstrate that although the central clock in mice always couples to external light cues, acute jet-lag promptly disrupts the coordination of the central and peripheral clocks, whereas chronic jet-lag abolishes the peripheral clock function in the absence of circadian gene mutations.

### Sympathetic Signaling Is a Mitogenic Signal

**Figure 5:**
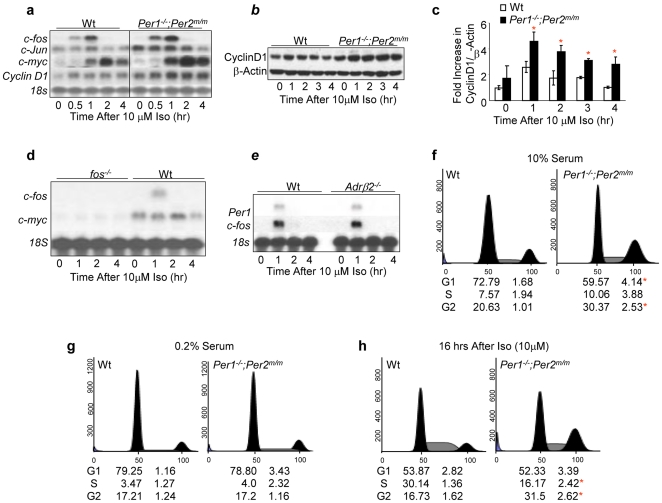
Hyperplastic Growth of *Per*-mutant Osteoblasts Is Extracellular Signal-dependent. (***a***). Northern blot analysis of *Ap1*, *c-myc* and *Cyclin D1* induction in 10 µM iso-treated wt and *Per1^−/−^*;*Per2^m/m^* preosteoblasts. (***b***) Western blot analysis of Cyclin D1 expression in 10 µM iso-treated wt and *Per1^−/−^*;*Per2^m/m^* preosteoblasts. The level of β-Actin in each sample is detected as a loading control. (***c***) A summary of Cyclin D1 induction from three independent Western blot analyses described in (***b***) (±SEM). (***d***) Northern blot analysis of *c-myc* induction by 10 µM iso in wt and *c-fos* ^−/−^ preosteoblasts. (***e***) Northern blot analysis of *Per1* and *c-fos* induction by 10 µM iso in wt and *Adrβ2* ^−/−^ preosteoblasts. The ratios of G1, S, and G2 phase cells of wt and *Per1^−/−^*;*Per2^m/m^* calvarial osteoblasts as determined by flow cytometry analysis under the normal growth condition (10% serum in culture media) (***f***), the serum starved condition (0.2% serum in culture media) (***g***), and at 16 hours after treatment with 10 µM iso (***h***). *Per1^−/−^*;*Per2^m/m^* osteoblasts show an increase in G2 phase in less than 16 hours after iso treatment, whereas wt osteoblasts only show an increase in S phase but not in G2 phase at the same time. The numbers below each histogram summarizes three independent flow cytometry analyses (±SEM). Asterisks indicate statistically significant differences.

To investigate whether SNS signaling stimulates cell cycle progression, we treated wt and *Per1^−/−^*;*Per2^m/m^* calvarial osteoblasts (preosteoblasts) with isoproterenol (iso), a synthetic agonist for ADRβ2 and β3 expressed on the preosteoblast cell membrane [54], [55]. We found that iso induced *Ap1*, *c-myc* and *cyclin D1* mRNAs as well as Cyclin D1 protein in both wt and *Per1^−/−^*;*Per2^m/m^* preosteoblasts, but the level of *c-myc* and *cyclin D1* induction was significantly elevated in *Per*-mutant cells (Fig. 5a–c). The induction of *c-myc* by iso was *c-fos*-dependent (Fig. 5d). Iso activated *Per* and *c-fos* in preosteoblasts via ADRβ3, or both ADRβ2 and ADRβ3 since *ADRβ2^−/−^* preosteoblasts showed an intact *Per1* and *c-fos* induction by iso (Fig. 5e). We then studied whether iso stimulates osteoblast proliferation. We found that *Per1^−/−^*;*Per2^m/m^* osteoblasts showed a higher G2 cell population compared to wt controls when grown in media containing 10% serum (Fig. 5f), which indicates that these cells had an accelerated rate of proliferation. However, under serum-starved conditions, wt and *Per1^−/−^*;*Per2^m/m^* osteoblasts arrested equally well (Fig. 5g). Iso induced cell cycle progression in both wt and *Per1^−/−^*;*Per2^m/m^* preosteoblasts but iso-treated *Per1^−/−^*;*Per2^m/m^* osteoblasts needed less time to progress from G1 to G2 phase compared to wt controls (Fig. 5h). The accelerated proliferation of *Per1^−/−^*;*Per2^m/m^* osteoblasts correlated with the over-induction of *c-myc* and Cyclin D1 by iso in these cells (Fig. 5a–c). Thus, hyperplastic growth of *Per1^−/−^*;*Per2^m/m^* osteoblasts is dependent on extracellular mitogenic signals.

### The SNS Activates ATM-p53 Signaling to Prevent Myc Oncogenic Activation

**Figure 6:**
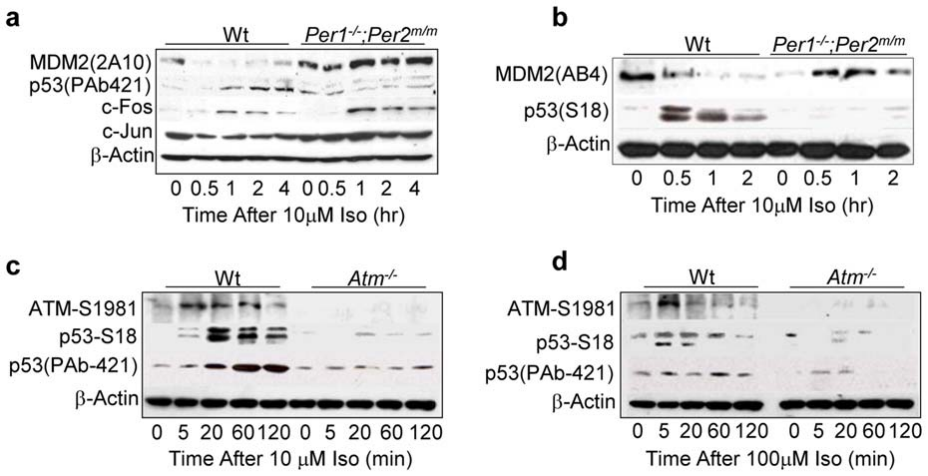
Sympathetic Signaling Activates p53 via ATM. (***a***) Western blot of p53, MDM2, c-FOS and c-JUN in 10 µM iso-treated wt and *Per1^−/−^*;*Per2^m/m^* osteoblasts using anti-p53 PAb421, MDM2-2A10, c-FOS and c-Jun antibodies. (***b***) Western blot of p53 and MDM2 in 10 µM iso-treated wt and *Per1^−/−^*;*Per2^m/m^* osteoblasts using anti-p53-S18 and MDM2-AB4 antibodies. (***c***) Western blot analysis of ATM-S1981 and p53 in 10 µM iso-treated wt and *atm^−/−^* osteoblasts using anti-ATM-S1981, p53-S18 and p53-PAb421 antibodies. (***d***) Western blot analysis of ATM and p53 in 100 µM iso-treated wt and *atm^−/−^* osteoblasts.

Since p53 plays a key role in preventing Myc oncogenic activation [48], we studied whether iso also induces p53 expression in preosteoblasts. We found that p53 was induced by iso with kinetics similar to AP1 transcription factors in wt osteoblasts. However, iso-treated *Per1^−/−^*;*Per2^m/m^* osteoblasts showed AP1 overexpression in the absence of p53 induction (Fig. 6a). The expression of p53 is mainly controlled by its interaction with the E3 ubiquitin ligase MDM2 *in vivo* [56], [57]. Thus, we examined MDM2 expression in iso-treated preosteoblasts. We found that MDM2 was suppressed by iso with the same kinetics as p53 induction in wt osteoblasts, but remained high in *Per1^−/−^*;*Per2^m/m^* osteoblasts (Fig. 6a). The MDM2-p53 interaction is best studied in γ-radiation response, which activates protein kinase ATM that phosphorylates p53 at Ser18 (S18) and MDM2 at Ser395 (S395) in mice. The phosphorylation at these two sites blocks MDM2-p53 interaction, leading to MDM2 autoubiquitination and p53 induction [58]. Since we used an anti-MDM2 2A10 antibody raised against a MDM2 C-terminal region containing the S395 residue, the interaction of 2A10 with MDM2 could be blocked by MDM2 S395 phosphorylation [58]. Thus, we studied MDM2 and p53 expression in iso-treated preosteoblasts using an anti-p53 S18 antibody and an anti-MDM2 AB4 antibody that interacts with MDM2 at the N-terminal region. We found that these two antibodies detected a similar rate of p53 induction and MDM2 degradation in wt osteoblasts as shown by the p53 PAb421 and MDM2 2A10 antibodies but failed to detect p53 S18 induction and MDM2 decrease in *Per1^−/−^*;*Per2^m/m^* osteoblasts (Fig. 6b). We then studied p53 induction in iso-treated wt and *Atm^−/−^* preosteoblasts. We found that in wt osteoblasts, ATM was activated by iso, as shown by ATM S1981 phosphorylation [59], in an iso dose-dependent manner and that the duration and the levels of ATM activation correlated with the level of p53 S18 phosphorylation and total p53 accumulation. In contrast, *Atm^−/−^* osteoblasts that lacked p53 S18 phosphorylation failed to show p53 induction by iso (Fig. 6c–d). Together, our findings indicate that the SNS activates p53 via ATM in a peripheral clock-dependent manner.

### SNS-controlled p53 Signaling Plays a Key Role in Tumor Suppression

**Figure 7:**
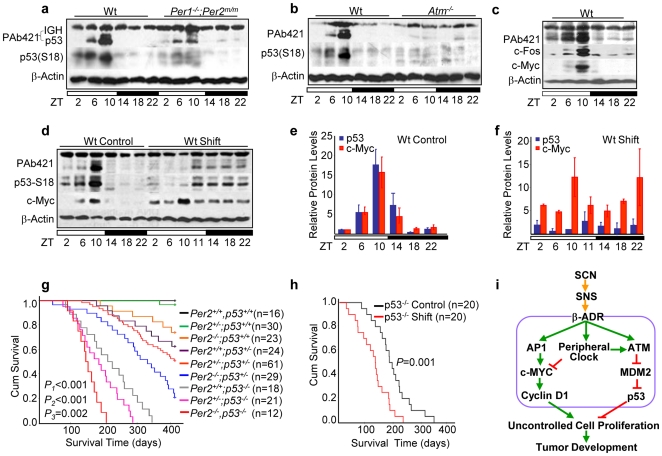
Circadian Expression of p53 Suppresses Tumor Development. Western blots of p53 expression in (***a***) the wt and *Per1^−/−^*;*Per2^m/m^* and (***b***) the wt and *atm^−/−^* thymuses over a 24hr LD cycle (The PAb421 antibody also detected immunoglobulin heavy chain (IGH) in total protein extracts from the thymus, which run above p53 as a 64 kDa band). (***c***) Western blot of p53, c-Fos and c-Myc in thymuses of wt mice over a 24hr LD cycle. (***d***) A representative Western blotting of p53 and c-Myc expression in the thymus of control wt mice and wt mice treated with one cycle of jet-lag (shift). A summary of three independent Western blot analyses described in (***d***) for control (***e***) and jet-lagged (***f***) wt mice (±SEM). (***g***) The Kaplan-Meier survival curves of mice with different copies of *p53* and *Per2* (*p*1: *p53^+/+^*;*Per2^−/−^* vs. *p53^−/−^*;*Per2^−/−^* littermates, *p*2: *p53^+/+^*;*Per2^−/−^* vs. *p53^+/−^*;*Per2^−/−^* littermates, and *p*3: *p53^−/−^*;*Per2^+/+^* vs. *p53^−/−^*;*Per2^−/−^* littermates). Median survival times in weeks (95% CI) are 55.8 weeks (52.03–59.58) for *p53^+/+^*;*Per2^−/−^* mice, 44.4 weeks (38.8–50) for *p53^+/−^*;*Per2^−/−^* mice, 28.9 weeks (25.3–32.56) for *p53^−/−^*;*Per2^+/+^* mice, and 21 weeks (18.1–23.9) for *p53^−/−^*;*Per2^−/−^* mice. (***h***) The Kaplan-Meier survival curves of control and jet-lagged *p53^−/−^* mice. Median survival times in weeks (95% CI) are 27.5 weeks (23.3–31.6) for control and 17.6 weeks (14.1–21.1) for jet-lagged *p53^−/−^* mice. (***i***) A model for circadian control of tumor suppression.

We then studied p53 expression and S18 phosphorylation *in vivo*. We found that the expression of total p53 and p53 S18 phosphorylation followed a coupled circadian rhythm over a 24hr period and peaked around ZT10 in most wt thymuses studied. In *Per1^−/−^*;*Per2^m/m^* thymuses, p53 S18 phosphorylation was almost undetectable, and the total p53 level was significantly dampened over a 24hr period (Fig. 7a). In *Atm^−/−^* thymuses, the circadian rhythm of p53 expression and p53 S18 phosphorylation was completely abolished (Fig. 7b). In addition, in 24hr LD cycles, the circadian expression of p53 was coupled with c-Fos and c-Myc in all wt mouse tissues studied including thymus, liver, kidney and white adipose tissues (Fig. 7c and data not shown).

We next studied p53 and c-Myc expression in the thymus of jet-lagged wt mice and found that one cycle of jet-lag was sufficient enough to uncouple p53 and Myc signaling, inhibit p53, and stimulate Myc expression (Fig.7d–f). To further study the role of p53 in clock-controlled tumor suppression, we crossed *p53^−^* ^/−^ mice with *Per2^−/−^* mice that have deficient peripheral clocks and overexpress *c-myc* [19]. We found a *Per2* and *p53* dose-dependent reduction in survival time in mice due to increased tumor development. Complete loss of *Per2* function reduced the average life span of *p53^−^* ^/−^ mice from 6 months to 4 months (Fig. 7g) [60]. We then treated *p53^−^* ^/−^ mice with jet-lag and found that jet-lagged *p53^−^* ^/−^ mice showed increased liver and salivary gland hyperplasia, kidney failure, and accelerated tumor development compared to untreated control *p53^−/^* ^−^ mice. Lymphoma and osteosarcoma were identified among jet-lagged *p53^−^* ^/−^ mice, with 10% of tumors being osteosarcoma (Fig. 7h and data not shown).

## Discussion

We have found that circadian gene-mutant mice share common aging and cancer-prone phenotypes although these symptoms may be more evident at different times during lifespan or under different physiological conditions in different mouse models. Disruption of circadian rhythm abolishes the peripheral clock which induces a tumor spectrum in wt mice similar to that found in circadian gene-mutant mice. These findings demonstrate that tumor suppression *in vivo* is a clock-controlled physiological function but not a non-clock function of a specific circadian gene.

Epidemiological studies have shown that disruption of circadian rhythm increases the risk of breast, prostate, endometrial, ovarian, lung, colon and pancreatic cancers, as well as non-Hodgkin's lymphoma, osteosarcoma and hepatocellular carcinoma in humans [7], [8], [9], [10], [11], [12], [40], [41], [61], [62], [63], [64]. The role of circadian dysfunction in mammary tumor development in rodents has also been well-documented [1], [2], [3], [4], [5]. Our studies show that chronic jet-lag also increases the risk of ovarian, kidney, intestinal and pancreatic cancers, osteosarcoma, lymphoma and hepatocellular carcinoma in mice. Together, these studies suggest that the mechanism of clock-controlled tumor suppression is conserved during evolution.

Irradiated and jet-lagged male wild-type mice show relatively lower tumor incidences and later onset of tumors than their female littermates. However, we did not find a significant gender-dependent difference in the time of tumor onset and total tumor incidence among irradiated and jet-lagged circadian gene-mutant mice. Although a larger sampling size may show a gender-dependent tumor development in irradiated and jet-lagged mutant mice, our studies indicate that gender-independent mechanisms are also involved in clock-controlled tumor suppression. Recently, nonsexual hormones leptin and growth hormones have been linked to the development of colon and breast cancers as well as non-Hodgkin's lymphoma in humans [65], [66], [67]. A role for SNS dysfunction in the development and/or progression of various human cancers has also been reported, which include prostate, breast, pancreatic, colon and ovarian cancers, glioma, neuroblastoma, osteosarcoma, hepatocellular carcinoma, cholangiocarcinoma and non-Hodgkin's lymphoma [68], [69], [70], [71], [72], [73], [74], [75], [76], [77], [78]. These findings in conjunction with our own studies indicate that circadian dysfunction of sympathetic activity could promote tumor development independently and/or synergistically with sex hormones.

The direct and indirect neuronal control of SNS by the central clock and the multi-synaptic control of peripheral tissues by the SNS have been intensively studied [43], [46]. Our studies demonstrate for the first time that *in vivo*, the rhythmic SNS signaling is a key central-peripheral timing mechanism that couples cell proliferation and tumor suppression with mammalian daily physiology. Such circadian control provides a gender-independent mechanism for tumor suppression. The identification of *Ucp1* as an endogenous reporter for both central and peripheral clock activities in our studies also provides a powerful tool for analyzing and comprising the status of endogenous circadian homeostasis which could not be evaluated by behavioral studies due to the light masking effect on the central clock.

Previous studies have shown that *Per*-mutant mice are cancer-prone, whereas *Cry1^−/−^*;*Cry2^−/−^* mice are deficient in cell proliferation in the first 72 hours of liver regeneration [19], [22], [24], [29]. A similar deficiency in liver regeneration has been reported for mice lacking the nuclear receptor FXR which are also prone for spontaneous hepatocellular carcinoma [79], [80]. These findings, along with our own studies, suggest that cell proliferation is differentially controlled under different physiological conditions *in vivo*. Using the central clock-SNS-peripheral clock axis as a model system, we propose that the central clock-controlled SNS signaling generates a coupled AP1, peripheral clock, and ATM activation. The activation of AP1 leads to *myc*-induced cell cycle progression, while the activation of the peripheral clock inhibits *myc* overexpression and is required for ATM activity. ATM then induces p53 to prevent Myc oncogenic signaling by blocking p53-MDM2 interaction. Disruption of circadian rhythm desynchronizes the central clock-SNS-peripheral clock axis which suppresses peripheral clock and peripheral clock-dependent ATM-p53 signaling but has no effect on *c-myc* activation. Together, these events lead to Myc oncogenic activation that promotes genomic instability and tumor development (Fig. 7i). Our model suggests that the circadian clock plays a dual role in cell cycle control and it suppresses tumor development by controlling the homeostasis but not the inhibition of cell proliferation.

Our finding of the sympathetic control of ATM-p53 signaling indicates that the induction of p53 occurs as an integrated part of mammalian daily physiology *in vivo*. This finding has important therapeutic implications. Recent studies indicate that cancer chronotherapy improves therapeutic index [81]. This may, at least in part, be explained by the fact that chronotherapy is usually applied during the sleeping phase when the endogenous p53 level is high, allowing host tissues to better tolerate genotoxic insults that induces p53 and p53-dependent apoptosis in somatic cells expressing low levels of p53. We have previously reported that wt thymocytes are less protected from radiation-induced apoptosis if mice are irradiated at ZT2 or ZT18 when the endogenous p53 level is low compared to thymocytes irradiated at ZT10 when the endogenous p53 level is high (Fig. 7a–c) [19]. Further study of clock-controlled ATM-p53 signaling is important for the development of novel strategies for cancer prevention and treatment.

## Materials and Methods

### Animal Maintenance

Animal experiments were approved by the Baylor College of Medicine Institutional Animal Care and Use Committee. Research was conducted in compliance with the Animal Welfare Act Regulations and other Federal statutes with regard to animals and experiments involving animals and adheres to the principles set forth in the Guide for Care and Use of Laboratory Animals, National Research Council, 1996. Mice were generated by heterozygous breeding of C57BL/6 inbred strains of *Bmal1^+/−^* [50], *Cry1^+/−^*;*Cry2^+/−^* [82], *Per1^+/−^*;*Per2^+/m^* [52], *Per2^+/−^* [19], *Per2^+/−^*;*p53^+/−^* and *p53^+/−^* [60] mice, and 129/C57BL6 hybrid strain of *Atm^+/−^* mice [83], and maintained under standard pathogen-free conditions of 2–5 per cage at 21°C–23°C with a humidity of 50%–70%, an air-flow rate of 15 exchanges/hr and in 24hr L/D cycles (light on at ZT0 and off at ZT12) if no specific light/dark conditions were described in the text.

### Survival Curve Studies

Similar numbers of male and female mice of each genotype were used for all survival curve studies. Mice were monitored 2–3 times a week following the five Morton and Griffiths aspects of animal conditions as a guide. Terminally-ill mice showed multiple symptoms including lack of food-intake, difficulty in standing, walking and breathing, visible abnormal growth at localized body parts, dramatic loss of body weight, ulcerated skin infections that did not respond to antibiotic treatment, and bleeding from the mouth, vagina, and/or anus, etc. These mice were sacrificed before death and all their vital organ/tissues were inspected. The major organs and tumors were isolated and processed for histological analysis. More than 95% of terminally-ill mice identified were diagnosed with grave pathological conditions including metastasizing tumors that had caused extensive damage of affected tissues, severe hyperplasia of reproduction organs or renal failure that would have caused death. The estimated time of natural death for these mice was ≤1 or 2 days from the time of euthanasia.

### Mouse Tissue and Urine Sample Collection

For collecting mouse tissues and urine samples in DD cycles, mice were maintained in standard LD cycles for two weeks and then placed in 24hr DD cycles for two days before being sacrificed for sample collection. For collecting mouse tissue samples from jet-lagged mice, mice were placed in 24hr LD cycles for two days after jet-lag prior to tissue isolation at indicated ZT times.

### Jet-lag

Mice were shifted every 3 days from 6 weeks of age between a 24hr LD cycle of 6:00am to 6:00pm light/6:00pm to 6:00am dark and a 24hr LD cycle of 10:00am to 10:00pm dark/10:00pm to 10:00am light.

### γ-Radiation

Mice were irradiated at 6 weeks of age at ZT10 with a single dose of 4Gy (16.8 cGy/sec) sublethal γ-radiation in a cesium-137 Gammacell.

### Histological Analysis

Tissue and tumor samples were fixed in formalin. Paraffin sections were prepared and stained with haematoxylin and eosin following standard procedures. All tumor types were confirmed by histological diagnosis.

### RNA and Protein Analysis

Northern and Western blot analyses were performed following the standard procedures as described previously [19]. RNA expression was detected by Northern blot analysis and quantified using a Molecular Dynamics Storm 860 PhosphorImager/Fluorima. Protein expression detected by Western blot analysis was quantified using the ImageJ program.

### Antibodies

Anti-MDM2 2A10 (Calbiochem), PAb421 (Oncogene), anti-c-Fos (Santa Cruz), anti-c-Jun (Santa Cruz), anti-CREB S133 (Santa Cruz), anti-c-Myc (Santa Cruz), anti-ATM-S1981 (Calbiochem), anti-p53-S18 (R&D Systems), anti-MDM2 AB4 (Calbiochem), anti-MDM2-H122 (Santa Cruz) and anti-β-Actin (Sigma).

### Cell Culture

Calvarial osteoblasts (preosteoblasts) were isolated, cultured, and treated with iso (Sigma, final conc. 10µM or 100µM) as described previously [29].

### Flow Cytometry

Cells were fixed in 70% ethanol, incubated with PBS containing 50µg/ml propidium iodide, 0.2% Tween 20, and 1 mg/ml RNase at 4°C overnight, and then analyzed by a Becton Dickinson FACScan flow cytometer using CellQuest software (Becton Dickinson) as described previously [19], [29].

### Urine Catecholamine Assays

Urine norepinephrine and epinephrine levels were measured using the Catecholamine Research Biotrak Assay System (Amersham) by TLC following manufacturer's instructions [29].

### Statistical Analysis

All survival curves were plotted using the Kaplan-Meier survival analysis software. Other statistical analysis was assessed by Student's two-tailed *t*-test. Values were considered statistically significant at p<0.05.

## Supporting Information

Figure S1 Neoplastic Growth and Tumor Development in Circadian Gene-mutant Mice. (a) Uteri from untreated 7-week old wt (Cry1+/+;Cry2+/+), Cry1−/−;Cry2−/−, and Per1−/−;Per2m/m mice. (b) Uteri from an untreated 60-week old wt (Per1+/+;Per2+/+) and age-matched irradiated/jet-lagged Cry1−/−;Cry2−/− and Per1−/−;Per2m/m mice. (c) Representative pictures of lymphomas developed in the chest cavities of irradiated/jetlagged Bmal1−/−, Bmal1+/−, Cry1−/−;Cry2−/−, Per2−/− and Per1−/−;Per2m/m and wt (Per1+/+;Per2+/+) mice. Histological slides showing (d) over-distended seminal vesicles filled with seminal fluid in an irradiated/jet-lagged Per2−/− mouse, (e) ulcerative necrotizing dermatitis of an irradiated Cry1−/−;Cry2−/− male mouse, (f) osteosarcoma in an irradiated/jet-lagged Cry1−/−;Cry2−/− mouse, and lymphoma in (g) the salivary gland of an irradiated Per1−/−;Per2m/m mouse, (h) the lung and (i) skeleton muscle of irradiated Per2−/− mice, and (j) the ovary, (k) kidney and (l) liver of irradiated/jet-lagged wt mice.
(4.18 MB TIF)

Figure S2 Sympathetic Control of Gene Expression. (a) Western blot analysis of the activation of CREB in the thymus of wt mice over a 24hr LD cycle using an anti-CREB S133 antibody. (b) A summary of CREB activation detected from three independent experiments as described in (a) (±SEM). (c) CREB is activated by iso in both wt and Per1−/−;Per2m/m osteoblasts. (d) A summary of Bmal1 mRNA expression in BAT of untreated (Control) and jet-lagged (Shift) wt mice from 3 independent experiments (±SEM). (e) A summary of Per2 mRNA expression in BAT of untreated (Control) and jet-lagged (Shift) wt mice from three independent experiments (±SEM).
(0.30 MB TIF)
