# Supplementary figures and images for: Disrupting Circadian Homeostasis of Sympathetic Signaling Promotes Tumor Development in Mice
Source: PLoS One. 2010 Jun 7;5(6):e10995. doi: 10.1371/journal.pone.0010995 (PMC2881876; doi:10.1371/journal.pone.0010995)

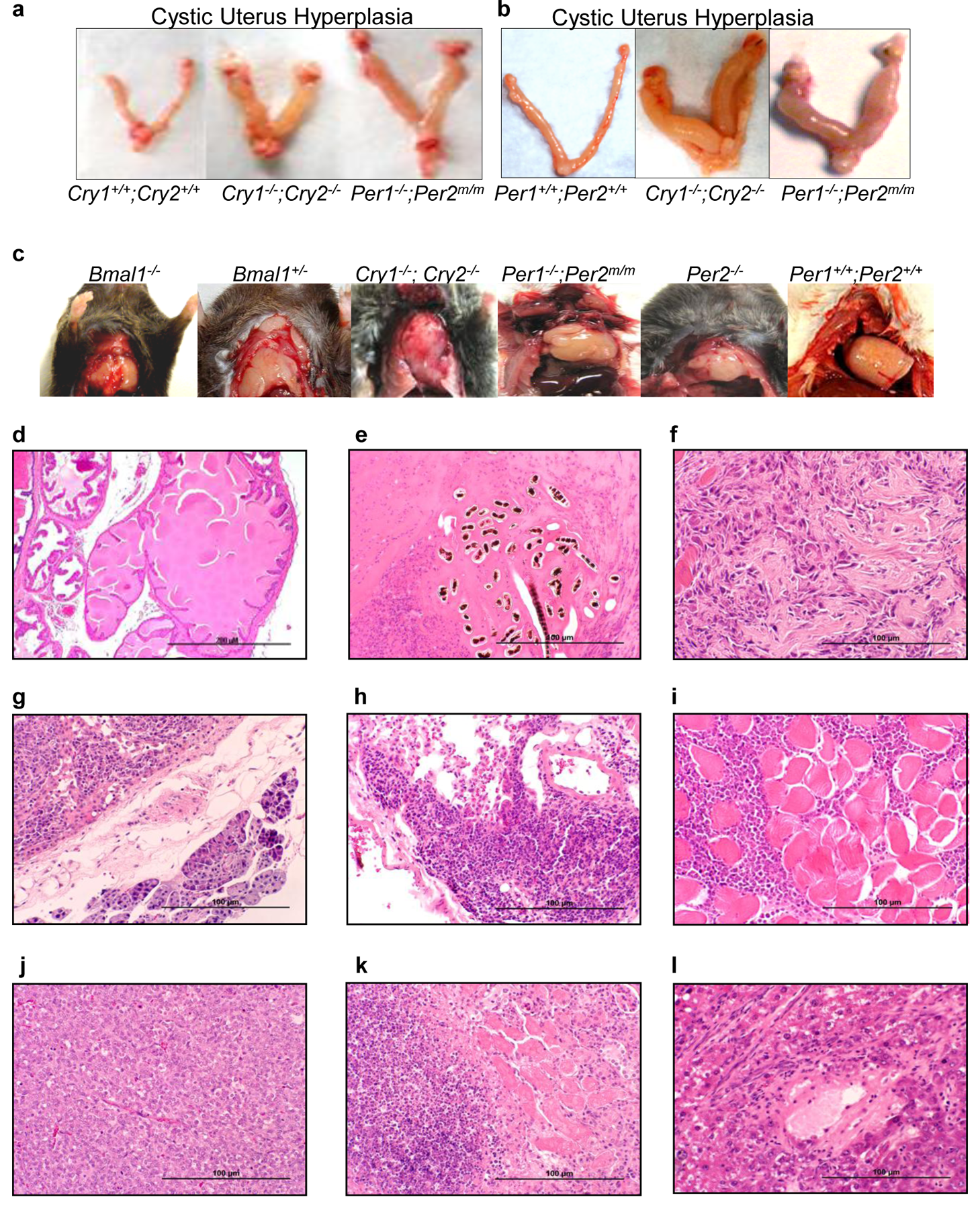

Supplement: Figure S1 — Neoplastic Growth and Tumor Development in Circadian Gene-mutant Mice. (a) Uteri from untreated 7-week old wt (Cry1+/+;Cry2+/+), Cry1−/−;Cry2−/−, and Per1−/−;Per2m/m mice. (b) Uteri from an untreated 60-week old wt (Per1+/+;Per2+/+) and age-matched irradiated/jet-lagged Cry1−/−;Cry2−/− and Per1−/−;Per2m/m mice. (c) Representative pictures of lymphomas developed in the chest cavities of irradiated/jetlagged Bmal1−/−, Bmal1+/−, Cry1−/−;Cry2−/−, Per2−/− and Per1−/−;Per2m/m and wt (Per1+/+;Per2+/+) mice. Histological slides showing (d) over-distended seminal vesicles filled with seminal fluid in an irradiated/jet-lagged Per2−/− mouse, (e) ulcerative necrotizing dermatitis of an irradiated Cry1−/−;Cry2−/− male mouse, (f) osteosarcoma in an irradiated/jet-lagged Cry1−/−;Cry2−/− mouse, and lymphoma in (g) the salivary gland of an irradiated Per1−/−;Per2m/m mouse, (h) the lung and (i) skeleton muscle of irradiated Per2−/− mice, and (j) the ovary, (k) kidney and (l) liver of irradiated/jet-lagged wt mice. (4.18 MB TIF) [file pone.0010995.s001.tif]

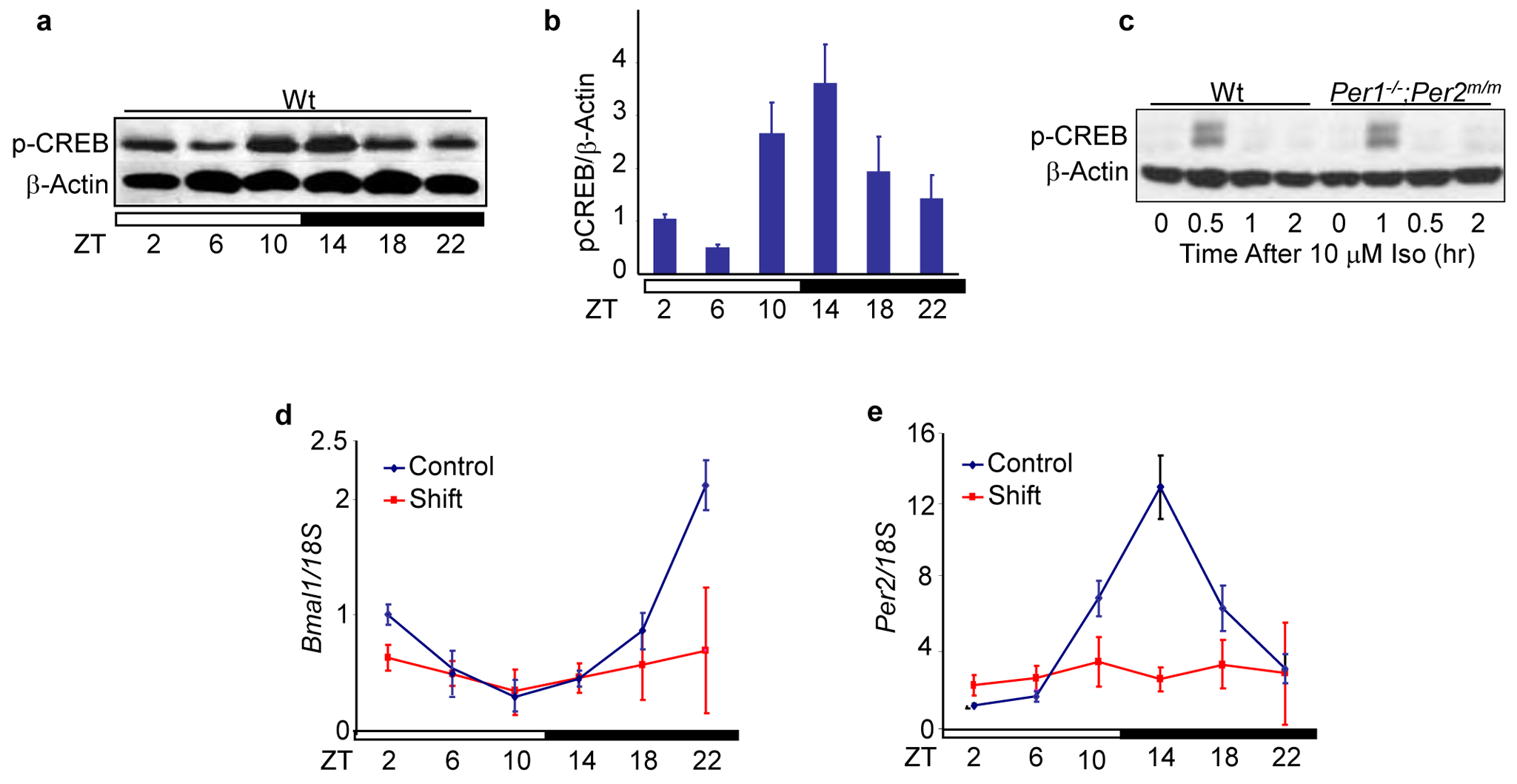

Supplement: Figure S2 — Sympathetic Control of Gene Expression. (a) Western blot analysis of the activation of CREB in the thymus of wt mice over a 24hr LD cycle using an anti-CREB S133 antibody. (b) A summary of CREB activation detected from three independent experiments as described in (a) (±SEM). (c) CREB is activated by iso in both wt and Per1−/−;Per2m/m osteoblasts. (d) A summary of Bmal1 mRNA expression in BAT of untreated (Control) and jet-lagged (Shift) wt mice from 3 independent experiments (±SEM). (e) A summary of Per2 mRNA expression in BAT of untreated (Control) and jet-lagged (Shift) wt mice from three independent experiments (±SEM). (0.30 MB TIF) [file pone.0010995.s002.tif]
